# Ambiguous participation in older hospitalized patients: gaining influence through active and passive approaches-a qualitative study

**DOI:** 10.1186/s12912-016-0171-5

**Published:** 2016-08-24

**Authors:** Ingrid Nyborg, Kari Kvigne, Lars Johan Danbolt, Marit Kirkevold

**Affiliations:** 1 Institute of Health and Society, University of Oslo, Blindern, P.O. Box 1130, NO-0318 Oslo, Norway; 2 Innlandet Hospital Trust, Kyrre Grepps gate 11, N- 2819 Gjøvik, Norway; 3 Hedmark University of Applied Sciences, Department of Nursing, P.O Box 400, N-2418 Elverum, Norway; 4 Nord University, Department of Nursing and Health, Sandnessjøen, Norway; 5 Norwegian School of Theology, Majorstuen, P.O. Box 5144, N-0302 Oslo, Norway; 6 Director of The Center for the Psychology of Religion, Innlandet Hospital Trust, P.O. Box 68, N-2312 Ottestad, Norway

**Keywords:** Patient participation, Older patients, Family, Next of kin, Geriatrics, Hospital ward, Phenomenological hermeneutics

## Abstract

**Background:**

Patient participation is required by law in Norway and in several western countries. Current participation ideology is based on individualism, which may conflict with the older generation’s commonly held values of solidarity and community. Hence, different values and ideologies may come in conflict when older patients receive treatment and rehabilitation in geriatric wards. Participation is a guiding principle in rehabilitation. Criteria for admission of older patients to geriatric wards are complex health problems, acute illness and/or acute physical and/or cognitive functional failure. The ideal is an active and engaged patient. The aim of the study was to describe the difficulties experienced by older patients on acute geriatric wards when involving themselves with their own treatment and care.

**Methods:**

In this qualitative study older patients were interviewed during hospitalization in geriatric wards and asked to tell about their experiences with participation. Data analysis was conducted using a phenomenological hermeneutic method.

**Results:**

The patients experienced difficulties in participating in decisions and care. They linked their difficulties to their own diminishing capabilities, and cited the ward’s busy schedule as a reason for abstaining from participation. However, despite their reservations, they did participate in decisions in different ways. Their participatory practices appeared ambiguous and they employed various strategies to put themselves in a position of influence. The most important of these involved their relatives. The patients delegated to family the tasks of seeking, receiving and giving information to the nurses and the staff, and, to some extent, for the dialogues with hospital staff about their needs and plan of care. The family appeared to accept the responsibility willingly.

**Conclusions:**

The patients addressed their difficulties by authorizing family members to act and participate on their behalf. This underlines the family’s important role in patient participation and the role that nurses and other staff must play in collaborating with the patient and their family to facilitate participation independently of the *patients’* performances of participation.

## Background

Patient participation in hospital wards may introduce alternate possibilities, solutions and decisions in treatment, care and rehabilitation because the patient perspective can raise different issues or questions than the professional perspective [1, 2]. The Norwegian health service is required by law to provide opportunities for patient participation [3]. The government’s preparatory writings stipulate that there must be genuine patient participation even if the patient suffers from hearing loss or cognitive impairment [4].

This study focuses on the difficulties of patient participation from the perspective of hospitalised older patients. The context was acute geriatric wards with a treatment and rehabilitation programme which included multimorbid older patients who suffer from complex health problems. The programme’s objective is to ensure that the patient’s health and functional capabilities improve. Within contexts like this, patient participation can be challenging albeit necessary and important [5–7]. One of the principles of rehabilitation is that the patient’s goals are imperative [2], and a good outcome requires an empowered patient in the form of a participating patient [2, 8, 9].

Several studies have explored patient participation among older hospitalized patients. One study conducted in a ward for frail older people related participation to activity of daily living [10]. Activities such as choosing which clothes to wear, where to sit, and what to do, were fundamental to the process of participation for older patients [10]. Thompson [11] related participation to decision making when he studied patients’ involvement in consultations, treatment and continuing care in the context of primary health care. Based on individual and group interviews, the patients’ desire to be involved was operationalized on five levels of patient-determined involvement: ^0)^ non-involvement, ^1)^ patients seeking and receiving information, ^2)^ patient information-giving, possibly dialogue, ^3)^ shared decision making, ^4)^ autonomous decision making [11–13]. Thompsons’s study excluded adults whom general practitioners deemed too ill or unable to participate [11], but the classification was considered appropriate in this study because it clarifies the relationship between information and decisions. By law, an individual’s ability to give and receive information must be taken into account when determining the appropriate level of participation [3].

Other studies on patient participation within hospitals point to disparities such as differing ideas of older patients and healthcare personnel with respect to what patient participation should entail [14–18]. Patients considered their own input to be related to their life situation while that of staff was related to diagnoses and medical matters [14, 15]. Healthcare professionals considered problems to be of a practical nature (assistance provided by community nurse), but the patients perceived them as existential in nature (grief caused by activity loss) [16].

Different values and ideologies may conflict when patients receive treatment and rehabilitation in geriatric wards. According to rehabilitation philosophy, the ideal is an active and engaged patient [2, 8, 9]. However, a systematic review on the experience of older people and their relatives in acute care settings shows that patients’ capacity for engagement and participation may be overlooked or reduced in such situations [19]. Current participation ideology is based on individualism, which may conflict with the older generation’s commonly held values of solidarity and community [20]. Older patients’ preferred ways of participation may not fit the contemporary idea of participation [20, 21]. Older people’s humbleness and gratefulness to the care system has been found to “outweigh” lack of information and participation at the cost of rejection of their own needs [22]. A slightly contrary suggestion was given by Foss [20] who found that older patients’ participation was subtle and discreet and involved finding strategies for interacting and initiating dialogue with healthcare personnel. The research reviewed above suggests that participation among older patients may be challenging and that we do not know enough about how older patients themselves understand and enact patient participation. The older patients’ positioning and ways of handling patient participation are not well explored. Consequently, this study concerns the difficulties experienced by older patients in the participation process and the actions they take and strategies they use to gain influence in spite of these difficulties.

### Aim

The aim of the study was to describe the difficulties experienced by older patients on acute geriatric wards when involving themselves with their own treatment and care.

## Method

### Design

The study had a qualitative design with a phenomenological hermeneutic approach based on Lindseth and Norberg’s method for studying lived experience by analysing interview texts [23]. This method was inspired by Ricoeur’s theory of interpretation [24], which highlights the interrelationship between epistemology and ontology, meaning that the interaction between the storytellers’ narratives and the researcher’s interpretation of those narratives create knowledge. The ontological part is justified in the theory’s linking of experience, understanding and self-understanding on the part of both the narrator and the interpreter, which grounds Ricoeur’s theory of interpretation in human existence [24]. Experiences, as such, are implicit in a situation and in the narrative about the situation [25].

The most radical move in this theory is the concept of distanciation which involves objectification of the text [24] in the sense that a text lives its own life separate from the narrator [26]. According to Ricoeur, “the truth is not hidden behind the text; it is disclosed in front of the text, when the interpreter meets the text” ([23], p 151). The interpretation always exists within the dynamics of the conjunction of interpretation and interpreter. When a text becomes familiar to the interpreter (the concept of appropriation), the entrance to the hermeneutic circle contains the dynamics of distance from and nearness to the text [23, 24].

### Context

The study was conducted in two Norwegian geriatric wards with six and twenty beds respectively. Criteria for admission for older patients were multimorbidity with complex health problems, acute illness and/or acute physical and/or cognitive functional failure. The rehabilitation and discharge planning process started immediately after hospitalization, in parallel with medical diagnostics and treatment, and interaction with the primary health service and relatives. The staff included geriatricians, geriatric nurses, nurses, nursing assistants and support workers, doctors, occupational therapists and physiotherapists with access to facilities on the wards.

### Participants

A purposive selection strategy was chosen for this study in order to include persons with experience with patient participation related to the hospital stay and to discharge planning. Patients acutely admitted to two geriatric wards, one at a small hospital and one at a university hospital, were invited to participate. Potential participants were identified by nurses who had been given this authority from the wards head nurses. Fifteen participants, five men and 10 women, aged 71–92 were included; see Table 1 for an overview. Age-related sensory impairment, cognitive restrictions and motor impairment were not considered obstacles to inclusion, but the patients would have to be able to participate in a qualitative interview. The participants received written and verbal information about the purpose of the study. It was emphasized that participation in the study was voluntary and that consent could be withdrawn at any time and without any kind of repercussion. All patients were ethnic Norwegians and they all had living relatives: five had spouses and a number of the participants had middle-aged children and children-in-law. All information in Table 1 was extracted from the patients’ narratives. All patients were ethnic Norwegians and they all had living relatives: five had spouses and a number of the participants had middle-aged children and children-in-law. All participants were old age pensioners and had been in paid employment of various kinds while of wage-earning age. Five patients suffered language and speech problems and a number of them were hard of hearing.

**Table 1:** Participants

Patient	Age	Gender	Acute illness/acute function failure	Social and physical surroundings. Assistance.
A	84	F	Fall. Vertigo.	Widowed. Small flat. Assistance provided by daughter.
B	73	F	Pneumonia	Married. Detached house. Assistance provided by husband and son.
C	71	M	Fall	Widower. Detached house. Private cleaner.
D	82	F	Visual disturbances	Widowed. Detached house. Assistance provided by daughter. Visited by community nurse.
E	88	M	Fall	Married. Sheltered housing. Assistance provided by wife. Community nurse visits day and night.
F	80	F	Fall. Medication poisoning.	Widowed. Detached house. Assistance provided by daughter and son-in-law.
G	88	F	Fall	Widowed. Farmhouse. Assistance provided by son and daughter-in-law. Personal safety alarm, which is not in use.
H	83	F	Pain	Widowed. Detached house. Assistance provided by son.
I	88	F	Fall. Breathing difficulties.	Widowed. Summons her daughter as necessary.
J	72	F	Pain	Married
K	71	F	Fall	Married
L	82	M	Dejected. Dehydrated.	Widower. Single after recent break-up with partner.
M	83	M	Fall	Widower. Detached house. Assistance provided by son. Home help. Personal safety alarm, which is not in use.
N	92	F	Arthritis. Pain. Breathing difficulties.	Widowed. Assistance provided by son and his family. Private cleaner. Community nurse.
O	83	M	Acute worsening of known disease.	Married. Detached house.

### Data collection

The main author was an observer at the two geriatric wards for 40 and 50 h respectively, in order to gain knowledge about their context, procedures and patients, and to determine what our best approach would be. Interviews were conducted with fifteen patients in 2013 while they were hospitalised in one of the two wards. One informant was interviewed at the nursing home eight days after being discharged from hospital and one was interviewed at the hospital eleven days after having been discharged. Data was generated through interviews based on a narrative approach. The concept of participation was defined in a theme-based interview guide (Table 2) and the general direction was established by key documents [2, 3, 27] and earlier research [9, 28, 29].

**Table 2:** Theme-based interview guide

Theme	
Health and life situation	Can you tell me something about your own needs and wishes for your treatment here?
	Is there any information about yourself and your situation that you think it is important you tell the nurses and the staff about?
	What do you consider to be your own resources?
Experience of participation and interaction	How do you feel that the nurses and the staff relate to you?
	Does anybody ask what you want and need?
	Have you ever participated in and influenced your own treatment?
	Can tell me about a situation where you contributed to a decision being made about what should happen?
	What influence did you have in that situation?
	Did anybody ask about your knowledge and experience?
	Would you like something to be different?
Own objectives	What is important to you here at the hospital?
	What is important to you in planning for your discharge?
	Are you being asked what your wishes and objectives are?
	Can you give an example of having discussed your situation with the nurses and the staff?

Some patients talked coherently while others needed to be guided through the process by the researcher’s body language, comments and follow-up questions [23]. Some patients suffered from concentration deficit and some from hearing and speech impairments; sound and conversation amplification aids were used. When talking to patients with dysarthria, the researcher would constantly repeat small fragments of the narrative to ensure that the account was confirmed as they went along. Many explained that they easily lost their concentration and/or that their memory was poor, and some asked to be reminded where they had got to if they came to a halt in their narrative. If patients were tired out by the interview process, they were offered a break. The interviews ranged from 12 to 67 min. Three interviews lasted approximately 20 min, a grouping of four interviews lasted about 30 min and six interviews roughly 40 min. The interviews were transcribed verbatim.

To ensure trustworthiness, we used investigator triangulation. The interview with the second participant was conducted by the first and second author, and the fifth interview by the first author and a study nurse. Over time and on several occasions, the authors all together, individually or in pairs, took part in the data analysis (as described below), and all authors critically reviewed and discussed the interpretation of the results.

### Data analysis

Framed by the hermeneutic circle, the Ricoeurian concepts of distanciation, appropriation explanation and understanding suggest a suitable theory of interpretation for textual analysis of research interviews [23]. The interpretation process includes movements between distance and proximity, between the whole and the parts of the text, and between understanding and explanation. This research methodology aims to move from an understanding of what a text refers to towards an understanding of what a text is about [23, 26]. The textual analysis involved three stages [23]. The first stage was *naïve reading*: the transcription was read to form an immediate impression of what the patients’ experience was about. During the second stage, s*tructural analysis*, our immediate impressions were honed by splitting the text into shorter or longer units of meaning relating to the research question. The units were condensed into themes and further into subthemes. These are presented in the results section. The third stage, *comprehensive understanding,* is presented in the discussion section. This will provide critical commentary with reference to the research question, earlier research and relevant literature.

### Ethical considerations

The study was approved by the Regional Committee for Medical and Health Research Ethics (REC South East Ref. 2012/1598). The older patients were in a vulnerable position in that they were ill and dependent on medical assistance. It was agreed with nurses that they would attend to the patients soon after their interviews were concluded and they would engage in conversation with any patient who so wished. The researcher was aware of and asked to be kept informed of any such needs. One patient took up the offer.

## Results

Two themes and five subthemes emerged from the structural analysis. These are shown in Table 3. The results are presented below, with quotes provided in order to illustrate the themes. The interdisciplinary professionals are mostly referred to as “hospital staff” or “the nurses and the staff” because the professionals mainly consisted of nurses.

**Table 3:** An overview of the themes and subthemes

Theme	Subtheme
Abstaining from participation	Complying with the hospital culture
	It is easier to tell if they ask
	Lack of capability
Entrusting and delegating responsibility to relatives	Lacking information
	Relatives as mouthpieces

### Naïve understanding

Some patients had heard about patient participation without in any way having related this to themselves, their health or the hospital. They expressed themselves in terms of feeling secure and being taken care of, and stated that the hospital staff was helpful, cheerful and friendly. The stories of participation mainly referred to personal care activities. Most of the patients felt that participation otherwise would require something that they themselves had only scant possession of: capability. They nevertheless demonstrated a vigour that would appear to suggest a level of capability, although this capacity ranged from acting directly to influence their care to acting indirectly through family members dependent on the patients’ own resources in a particular situation. To cope with the difficulties of participation, they strategically delegated the interactions with the staff to their relatives, entrusting responsibility to them. Thus, patients initiated and achieved participation, and relatives became mouthpieces and the patients’ representatives.

### Abstaining from participation

The patients’ stories of participation referred to grooming and dressing, food and meals for which they took the initiative and were in charge, negotiating with the nurses and interacting with fellow patients. Patient L (male, 82), noted how having opportunities to choose was valuable, even when this choice was only one between the courses on a menu, while another patient referred to choosing one’s own clothing as one such simple factor.

Our decision to consider abstaining from participation as a reasoned choice was grounded in the patients’ way of reasoning in the stories they told. In explaining their reasons for not participating, they referred to their own shortcomings or the staff’s lack of time, while at the same time they defended the ward’s arrangements and communicated having adapted to them.

### Complying with the hospital culture

The patients did not find it unreasonable for the hospital staff to not ask their opinion or ask what they wanted. The patients maintained that when they were in hospital they had to comply with the routines on the ward. Patients seemed to see this as thoughtfulness and we interpreted that it was a valuable trait for adapting to hospital culture. On a related note, time was a crucial theme throughout the interviews. Patient D (female, 82) linked time and trust. She did not want any involvement, as there was not enough time to establish trust in the personnel. She wanted to “*go along with things as they are*”, and gave the limited time available as her reason for doing so. The patients did not want to be considered difficult patients. They were worried about ‘nagging’ the staff*,* as they sensed the staff were exceedingly busy. “*The staff here have far too much to do, and people are quite stressed at times*” (Patient K, female 71)*.* Only patient G (female, 88), who also experienced “*how horrible it is to be a nuisance*”, explained that it was hurtful not to be able to participate, and that she felt sad because of it. She accepted that the nurses’ timetable was the premise that restricted her participation, but she did voice a slight opposition:

“I suppose I’d really like to be more involved, but I can see how they run from one bed to the next, and after all, I’m not the only person here! There are hundreds of other people. So there. But I suppose it would be nice if they did have more of a chat with us. Maybe” (Patient G, female, 88).

Patient K (female, 71) took a more pragmatic view when she said that “*the staff needs to take a firm handle of the situation and make a decision*”. She did as she was told, also because the hospital staff was responsible for “*making this place go rou*nd” (Patient K, female 71).

### It is easier to tell if they ask

Although the patients noted that they were obviously free to ask questions, they did not report doing so. They provided no information to the staff without being asked. Patient I (female, 88) was concerned about who was responsible for identifying information needs, explained that she perceived the patient as a respondent who gives answers:

“I’m quite uncertain about who is responsible for providing information. Which groups these are, whether they have a general approach and whether they have made a template that safeguards each patient’s need for information” (Patient I, female, 88).

One patient claimed that the hospital staff asked the right questions because they knew how much time was available. Patients’ descriptions of waiting for the nurses to take the initiative to ask questions revealed their passive stance towards information and decision making. The patients refrained from “*ringing the bell*” even if they had questions they wanted to ask. Patient M (male, 83) made no calls himself, noting that the nurses would regularly attend to him waiting bedside, to tell and give information.

Patients stated that they did not initiate conversation with the nurses and the staff because they had not been asked any questions. Patient C (male, 71) told he had refrained from providing information that he believed was important for his treatment. He had pondered the fact that nobody had asked him, yet he had never volunteered the information. If the staff did not ask about something, it was clearly not important.

“No, I haven’t said anything. I was never asked. They don’t ask me that much about how I want things – they’ve got their schedule, after all. You know, they have their meal times and their routines and that, and we just have to go with it” (Patient C, male, 71).

Patient J (female, 72) asserted that patients who were “mobile” were able to ask questions whenever they wanted rather than being dependent on the nurses and the staff. She included wakefulness and awareness of situation as part and parcel of being “mobile” meaning both that she could move around and that she was lucid and cognisant:

“Then you are able to ask questions. But you need to be clear in the head. You need to know what’s going on. You can’t be dozy or very poorly” (J female, 72).

### Lack of capability

Besides cognitive skills and the ability to pay attention, as seen above, being a competent participator (or not) was associated with knowledge, advanced age, level of endurance and positioning strategies.

Patient I (female, 88) linked the terms memory and information to the concept of capability, and this again to dependency of hospital staff in matters of information. She was adamant that patients should receive the level of information that matched their capability, and assessing this was the responsibility of healthcare personnel.

“It is extremely important to keep those patients informed who are able to take the information in and remember it. The staff will have to take great care in assessing who is capable of taking in information and be entirely sure that the patient is able to take in what they are told” (Patient I, female, 88).

The patients did not consider themselves to be knowledgeable and they did not expect to be able to influence their own situation. They deferred to the expert’s medical knowledge of diseases and treatment, which enabled them to make the right decisions. Patients felt that the nurses and the staff would try to do what they felt was for the best in any given situation, and that was enough. Patient D (female, 82), who found that the doctors in the ward round talked above her head, said: “*which they are very welcome to do, for I understand perfectly well that they are more knowledgeable than I am*”. She acknowledged that she did in fact have first-hand knowledge about *herself*, but this did not mean that she could contribute with her own personal knowledge to the treatment provided for her.

Patient E (male, 88) explained that old age as a factor in itself, and the associated lack of energy, played a part in whether or not one was capable of participation in treatment and care. He stated that his intelligence was intact but that he had no endurance: “*We are a little disabled when it comes to keeping on top of what is happening*”. He believed that most elderly people’s capabilities were blunted by old age and that being old and ill has a real bearing on the intellect, irrespective of whether or not this is recognised by the patient. Patient N (female, 94) who was making audible efforts throughout the interview said she had no energy for co-determination in question of treatment. Nevertheless, she took a keen part in discussions about adjustments to her walker, which held all the objects she wanted to keep close at hand.

One way of demonstrating capability was to establish positioning strategies, and the patients’ aim seemed to be either to take part themselves or to secure the participation of their families. Patient H (female, 83) said she was forgetful, and that she had told her son to talk to the management if there was anything he wondered about. Before Patient K (female, 71) started a conversation with the nurses and the staff, she would need to make sure that the person she was talking to was receptive. She sensed and tested the ground and “*wait until they’re not feeling stressed*”. Being cheerful was, according to the patient, her greatest resource in her encounter with the health service, but “*it depends on who the Health Service is*”. She said she gave the nurses praise, and she reckoned in this manner to come in position of dialogue:

“It’s not to ingratiate myself, but it is really important that the people who work here have a good working environment. Really important. And then we strike up a relationship. For of course, that makes them notice me in return” (Patient K, female 71).

### Entrusting and delegating responsibility to relatives

With the exception of three of the youngest patients, all of the patients explained how they specifically involved their relatives and delegated their participatory role to them. Most of the patients considered themselves and their relatives as a “we”: a unit that was at their disposal. Some took a rather controlling attitude, some were quietly accepting of the situation, while others talked to their family about reasonable task sharing. Evidently the patients experienced taking part whether they themselves or their relative took action.

### Lacking information

In the context of narratives about medical examinations, diagnostic imaging and relocations between wards, reference was made to how patients failed to obtain information and how they involved their relatives on these occasions. Remarkably, the patients themselves generally never actively pursued the information. Evidently they wanted to obtain information either from the hospital staff or family members, or to have staff members ask questions for them to answer. Patient N (female, 92) stated that she greatly appreciated it when the doctor would sit at her bedside to talk to her.

Patient D (female 82) put her daughter in charge of seeking information even though the following excerpt shows language skills, a high level of knowledge, insight and clarity:

“I don’t think that this looks like a mini-stroke. It could be a small one, though. I’ve been a little feeble in my legs, that’s all. And in my arms. The flickering in my eyes has nothing to do with it, I believe. They said I was going to have x-ray to have my head examined. I don’t think they will see much in that picture, really. I haven’t heard so much about the results. I don’t have so much self-confidence that I can (pause)… I cannot say so much about it, then. But I’m glad to have my daughter. For support, that is. She is so sensible. Gentle and clever” (Patient D, female 82).

Patient J (female, 72) described how she came to a gastroscopy completely unprepared. She said that the examination was uncomfortable, although not difficult to go through, because the nurse “*taught me how to breathe instead of coughing*”. Other patients perceived the lack of information as more dramatic. Patient I (female, 88) described how she was helped by her daughter during the admission, but was subsequently wheeled off in her bed without being told where she was going. She stated that she was an obedient patient, but on this occasion she had lost her temper and taken her frustration out on the staff. She had become terribly disappointed in herself and apologized to the staff. Patient G (female, 88) had also experienced some frustration but had several family members for support.

“I wasn’t expected there, even though all those who come out of surgery go there. I was extremely frustrated. Nobody knew what was going to happen to me or where my bed would be. I didn’t bring much with me, but then I didn’t even know where my cell phone was. It was sorted out and after a while I was assigned to a room. I have some relatives, after all” (Patient G, female, 88).

### Relatives as mouthpieces

The patients’ accounts revealed that a total of 24 family members (one husband, two wives, one daughter retired from work, middle-aged daughters and sons still working, sons-in-law, daughters-in-law, grandchildren) had been involved with the admission procedure and during the patients’ stays in hospital. Patients who had relatives among the hospital staff considered it natural for them to assist with the communication vis-à-vis treatment providers. Some explained that a number of their closest relatives took turns being present at the hospital. The hope was that they would be able to influence the time of discharge, which for many was an important factor.

Patient O (male, 83) was very ill and had on two previous occasions been sent home too early. He now had a team of relatives – son, daughter and wife – who jointly handled the relationship with the hospital. Patient O (male, 83) explained that it was not important to him to be able to influence the treatment, but that there was one thing he definitely wanted to have a say in: he did not want to be discharged too early.

“But I suppose it’s the hospitals that decide, really. That’s what I think, anyway. For we have tried now, junior and myself and my daughter and my wife, to have the discharge date pushed as far into the future as possible, to make sure I recover as much as possible. I’ve hardly been out of bed yet, you know” (Patient O, male, 83).

Some had contacted their children prior to admission, and patient I (female, 88) who had speech problems, explained that she had informed the hospital reception on admission that her daughter, who was a nurse, should be involved rather than herself.

“Well, I suppose I was there, listening to everything that was said, so in a way I was involved, but as far as I was concerned, I felt they would be better off just talking to my daughter through her profession, and then the two of us could talk it through at leisure afterwards” (Patient I, female, 88).

Patient D (female, 82) maintained that while in hospital the patients would be consulted. She said it was the patients’ duty to inform the ward round how they were, and that she herself had handed over this duty to her daughter. “*And they keep asking. So I call my daughter over, as I want her to help me. I’m worried I won’t give the right answers, you know*” (Patient D, female 82).

## Discussion

Central to our discussion will be ambiguity, as a general characteristic of the older patients’ participatory practice, and the delegation of responsibility to relatives.

### Ambiguous participation

The patients who took part in this study revealed an ambivalent attitude to involving themselves with their own treatment and care. Their participation appeared ambiguous. They alternated between participating, rejecting their participatory role and delegating responsibility for this role to their relatives. The older patients linked their participatory difficulties to their own diminishing capabilities, and they cited the hospital staff’s busy schedule as a reason why they refrained from involving themselves. While the patients talked at great length about their impairment, they nevertheless demonstrated a vigour that would appear to suggest a level of capability: They alternated between a passive stance, meaning they took no actions (in direction of the nurses and the staff) to participate in their own care, and an active stance (in direction of their relatives) depending on their own resources in a particular situation. They participated, in terms of initiating, making choices, interacting and negotiating, in activities of daily living, which, according to previous research, matters the most by improving and maintaining function, giving older patients a sense of purpose and perceived higher quality of life [30]. Some used their strategic skills to position themselves for influence, which is consistent with earlier research [20, 31]. The reasons cited by patients to explain why they preferred “passive” actions, meaning taking part through a family member, included feeling they had ‘negative equity’ due to their advanced years, low level of endurance, impaired cognitive skills and lack of knowledge. All of these factors have been found in other studies as well [8, 9, 16, 28, 32, 33]. The staff timetable set the premise for (the restricted) participation, and the patients talked of time as something that hospital staff controlled, and which they took [8, 33] and gave [34].

In keeping with findings in previous research, the older patients were reluctant to be a nuisance [8, 20], but eager to participate [13]. As such this study showed both a personal involvement and patient-determined delegation of participation. Their desire to participate was evidenced by the fact that they delegated the responsibility to a proxy, and by the nature of the tasks they delegated. The patients would delegate to their families those tasks that they felt incapable of handling by themselves but that were necessary to exert influence and have a say in decisions [11]. The delegation might involve giving and receiving information at the time of hospitalization, communicating with the nurses and the staff during the hospital stay, attending when doctors were doing their rounds, and negotiating the date of discharge from hospital. In most delegated situations the patients’ physical presence did not make their participation less ambiguous.

When patient participation is accomplished within the family, and family members are clear representatives for patients when needed, this may suggest that a geriatric ward, in line with the patient perspective of rehabilitation philosophy, has a family-inclusive policy. In order to facilitate patient participation, the wards need to signal that they welcome diverse approaches to patient participation, and that family represents a basis for patient participation from the older person. Relationship-related approaches to care and participation in decision making is well described in literature [19]. Relatives, however, who according to the patients were virtual extensions of themselves and appeared to be at the patients’ disposal, probably missed an opportunity to participate on their own behalf in their own role as relative. According to national healthcare planning and goals for health policy, relatives must, beside to be the patient’s representative, be heard when it comes to their own needs [4].

The ambiguity expressed by the patients may be seen as indirect criticism of ward culture. Patients listed several shortcomings with respect to their circumstances and their (lack of) opportunities to influence and participate in their own care. On the other hand, the patients generally adjusted to the ward routines and many defended the ward arrangement. This is in line with previous research [33, 35]. In gerontology, the study of the ageing process, this may be seen as an age-related adaptive coping strategy: adjusting to one’s surroundings instead of trying to change them [36]. In geriatrics, the study of medical conditions and disease in the older [5–7], the patients’ ambiguous and fluctuating participation may illustrate the complexities that arise when old age coincides with complex health problems and acute illness. Whether the perceptive is different aspects of aging, or disease in older adults, the results showed that patients included family in their participatory practice.

### Patient-delegated participation through a family member

A review of research on engaging older adults in their transitional care says that a future challenge will be how to respond when patients choose not to become engaged [30]. Among older adults and other groups of vulnerable older adults, the engagement of family caregivers will be critically important [30]. When patients in this study actively addressed their own difficulties with participation in treatment and care, they would turn to their families. Patients delegated family members to participate on their behalf, and it appeared that the relatives responded willingly, seeking to collaborate with the hospital staff and thus compensating for the patients’ difficulties in seeking, receiving and giving information. Earlier research on how persons with dementia participate in decision making, based on Thompson’s taxonomy [11], has pointed to delegating decision making as a new category of patient involvement [37]. Ekelund et al. [38] too found that older persons may include the family in their way of being self-determined in decision making.

Patient-delegated participation occurred in this study as well. This result draws attention to the fact that older patients handled difficulties related to their own involvement and to the ways in which they did so. At admission to hospital and during their stay in hospital, the patients delegated the tasks of seeking, receiving and giving information, and to some extent, dialogue with professionals about their care. According to Thompson’s taxonomy, seeking and receiving information is considered as the elementary level for being able to take part in decision making, and second level (patient information-giving, possibly dialogue) enables the conversations between patient and clinicians [11, 37]. The patients sought assistance of their relatives in connection with difficulties such as fear of nagging the staff, being incapable of asking questions, fear of giving incorrect answers, speech impairment, and the need to be given sufficient time, peace and quiet to understand the information provided. When it came to the taxonomy’s third level, shared decision making [11], the results gave none examples with regard to the experienced difficulties of participation. The patient in this study who on two previous occasions had been sent home too early, had a team of family members who had tried to push the discharge date as far into the future as possible. The patient’s reflection was “*But I suppose it’s the hospitals that decide, really*”.

With regard to the experienced difficulties of participation, patient participation took place in a sphere which ran parallel to the “patient and the hospital staff” constellation. Patients relied on their family members, participating through them, which, again, points to the fact that participation of family represent a basis for the patient’s participation in treatment and care processes [19, 39]. This acceptance of responsibility by the family, as recounted by the patients in this study, is not well investigated in research when it comes to family members’ own perceptions of participation [12]. Nurses’ experiences of collaboration with family, however, have been explored, and one study called for a mandatory involvement of relatives at the time of admission and upon discharge with regard to information exchange [12, 39].

The experienced difficulties of participation may be discussed in light of the nurses and the staff’s approaches and communication. Collins et al. [40] examined doctors’ talk with patients and identified two decision making trajectories namely “unilateral” and “bilateral” approaches, and they suggested that the latter may improve patient participation in the context of health care. In the more “bilateral” approach, the decision making processes were enacted as an integral part of the communication in the consultations and took into account the patient’s contributions. The more “unilateral” approach structured the decision making processes somewhat independently of conversations with the patient” [40]. Our study may exemplify the more “unilateral” approach as far as the experienced difficulties were addressed the family and not the hospital staff. If the nurses and the staff facilitate participation in treatment and care as in the “bilateral” approach, the patients’ contributions may be addressed them and not the relatives.

Guided by the study of Collins et.al [40], Riva et al. [41] have described primary nursing approaches to patient participation in conversations on discharge planning. The study found that different communicative styles (named “reciprocal” and “individual”) exist, and that health professionals can adapt their communicative practices [41]. The reciprocal style is a more collaborative approach and relies on the nurses to initiate and moderate participation [41]. Besides accentuating that the nurses and the staff can adapt, initiate and moderate participation, our study highlight the importance of structuring patient participation independently of the patient’s capabilities and performance of participation. Older people enact the participant’s role in both active and passive ways. The fact that the hospital staff in this study mainly consisted of nurses points to the significant role of nurses in soliciting participation from older patients.

### Limitations

Interviews with people who suffer from attention deficit, psycho-motor tempo retardation, hearing impairment, language handicap or fatigue fail to fulfil all the criteria of an ideal qualitative interview [42] with short questions and spontaneous relevant answers that are rich in content [43]. The sheer quantity of interviews may have compensated somewhat for this weakness [42]. We have sought to communicate the results as transparently as possible.

## Conclusions

The patients delegated to family the tasks of seeking, receiving and giving information, and to some extent dialogues with the hospital staff. The patients’ participatory practice was ambiguous, alternating between active and passive approaches to influencing their care. The patients referred to their own impaired capabilities as an obstacle to participation, and cited the hospital staff’s busy schedules as the reason for their wait and see attitude. Still, they wanted to participate. They addressed their difficulties by authorising family members to act and participate on their behalf. This point to the family’s role in patient participation, but even more to the role that nurses and the staff play in collaborating with family adjusting participation independently of the *patients’* performances of participation.

It is essential that we take into account the fact that the family members in this study appeared to accept the responsibility of intervening on behalf of the patient willingly, and that family have the right to participate on their own behalf when affected by patient intervention. Further studies are required on how the relatives of older patients experience participation on their own behalf.
